# Exploring Non-Pharmacological Interventions as Part of Multimodal Management to Prevent Opioid Misuse in Adults Prescribed Opioids for Chronic Pain

**DOI:** 10.3390/jcm15114079

**Published:** 2026-05-25

**Authors:** Manar A. Alrashid, Maya S. Zumot, Salim Fredericks

**Affiliations:** 1 Department of Management, University of Liverpool, Liverpool L69 7ZX, UK; 2 East Lancashire Hospitals Trust, Blackburn BB2 3HH, UK; 3 Department of Biochemistry, Royal College of Surgeons in Ireland, Al Muharraq 15503, Bahrain

**Keywords:** opioid misuse, chronic pain, drug monitoring, CBT, patient education

## Abstract

In recent years, there has been an unprecedented upsurge in opioid prescriptions for pain management. Consequently, the widespread availability of these medicines has led to an increase in misuse and abuse. This has led to a greater number of overdose-related deaths. The high prevalence of drug misuse was born of multiple and complex societal factors. However, from a medical perspective, critical contributors to the dire consequences of the crisis have been the need for chronic pain relief, as well as mental health issues within communities. Chronic pain coupled with psychological distress exacerbates patients’ predicaments and thus further fuels the crisis. Anxiety and depression have bidirectional and complex relationships with pain. The somatic symptoms associated with anxiety potentially worsen pain, whilst pain emanating from a chronic condition worsens anxiety. The same relational dynamic applies to depression and pain. Thus, these psychopathological states may be major contributors to the opioid abuse epidemic. Thus, psychosocial management as a first-line treatment instead of starting with drug treatments seems an enlightened approach to this problem. Cognitive behavioral therapy (CBT) has been proven to be effective in managing specific symptoms associated with chronic pain. Similarly, patient education has been shown to be a viable alternative to drugs for certain aspects of chronic pain treatment. We consider that the opioid crisis could be addressed with a greater reliance and emphasis on non-pharmacological approaches to managing chronic pain patients. This mini-review examines non-pharmaceutical and monitoring-based interventions to reduce opioid misuse risk among adults prescribed opioids for chronic non-cancer pain. Studies were identified through PubMed/MEDLINE, Scopus, and Google Scholar using terms related to chronic pain, prescription opioid misuse, opioid use disorder, cognitive behavioral therapy, patient education, prescription drug monitoring programs, digital health, telehealth, and non-pharmacological interventions. Studies were included if they focused on adults with chronic pain who were prescribed opioids or at risk of misuse, and evaluated interventions aimed at reducing unsafe opioid use, misuse risk, or opioid-related harm. Evidence was synthesized narratively to identify key intervention approaches, limitations, and clinical implications.

## 1. Introduction

Over the past two decades, opioid prescriptions for pain management have surged, especially in the United States, making these drugs widely available and leading to increased misuse, abuse, and overdose-related deaths [1,2,3]. Multiple complex societal factors drive this crisis, but from a medical perspective, chronic pain relief needs and community mental health issues play critical roles [4,5].

Chronic pain is commonly defined as persistent or recurrent pain lasting longer than three months and extending beyond the expected period [6,7,8]. Opioid therapy can be considered for a select number of patients with moderate to severe pain. This includes cancer-related pain, those on palliative care, post-surgical or post-traumatic severe pain and a selected musculoskeletal or neuropathic pain where non-opioid therapies are ineffective or contraindicated [6,8]. Before starting opioid therapy, clinicians should assess pain intensity, functional impairment, previous treatment response, psychiatric comorbidity, substance misuse risk, concurrent sedative or alcohol use, and overdose risk. If prescribed, opioids should be initiated at the lowest effective dose, titrated cautiously according to analgesic response and functional improvement, and reviewed regularly for efficacy, adverse effects, aberrant drug-related behaviors, dependence, and continuing clinical need. In addition, non-opioid pharmacological treatments and non-pharmacological interventions should be incorporated into the management plan as part of a multimodal approach to chronic pain management. Patient-centered pain management should remain the priority, with clinicians supporting and encouraging opioid weaning where clinically appropriate [7,8].

Non-medical opioid use refers to the use of opioids without a prescription, in higher doses than prescribed, for longer than prescribed, or for reasons other than pain relief, such as recreational use or self-management of psychological distress. This pattern is more commonly reported among younger individuals, particularly those aged 25 years and below, who may obtain opioids from peers, family members, or illicit sources rather than through formal medical prescribing pathways [9].

Prescribed opioid use refers to the use of opioid medications under the direction of a healthcare professional for a recognized clinical indication, such as chronic pain. Among older adults, particularly those aged 26 years and above, opioid misuse may more commonly arise from medically prescribed opioids used for chronic health conditions associated with aging. In this group, misuse may include dose escalation, prolonged use beyond clinical need, or difficulty reducing opioids because of persistent pain, dependence, or inadequate monitoring [9].

Statistics often overlook older adults in opioid misuse data, as their misuse behaviors appear atypical [9]. Current pain management strategies rely primarily on pharmacological solutions, such as opioids for musculoskeletal pain and gabapentin for neuropathic pain, despite a 9% rate of opioid abuse among chronic pain patients [6,10]. These first-line treatments often fall short because they address only symptoms and fail to tackle the holistic and emotional aspects of chronic pain, which persist beyond tissue healing [7,8]. We urgently need more unconventional, comprehensive, and non-pharmacological approaches to manage chronic pain effectively. Comparing different regional approaches to opioid prescribing and pain management could offer valuable insights, helping to find optimal solutions to this crisis and improve patient outcomes.

Pharmacological approaches predominate in chronic pain management. In parallel, pharmacology predominates in the solutions served up to solve the current epidemic. Ironically, pharmacological treatments such as naltrexone or buprenorphine are employed to address this crisis [11]. Fighting fire with fire or, more precisely, pharmacology with pharmacology has proven not to be effective as the trend of opioid misuse continues to linearly increase, especially in older adults [12]. Drug abuse, like chronic pain, needs to be viewed comprehensively. Anxiety and depression have bidirectional and complex relationships with pain. The somatic symptoms associated with anxiety potentially worsen pain, whilst pain emanating from a chronic condition worsens anxiety [13]. The same dynamic applies to depression and pain. Thus, these highly prevalent psychological states may be major contributors to the opioid abuse epidemic [14,15]. Thus, psychosocial management as a first-line treatment instead of starting with drug treatments seems like an enlightened approach to the problem [16]. Hence, non-pharmaceutical interventions should be considered viable solutions to this problem. Cognitive behavioral therapy (CBT), patient education, and continuous drug monitoring need to be explored and used alongside or instead of drug-based treatments.

Non-pharmacological interventions are important in chronic pain management and may help reduce opioid use and misuse. However, the opioid crisis is complex and must be approached from multiple directions. While non-pharmacological strategies may help prevent opioid dependence, it is also important to consider individuals who are already experiencing opioid misuse or opioid use disorder. For these patients, Medication for Opioid Use Disorder (MOUD) plays an important role. Medications such as methadone, buprenorphine, and naltrexone have been associated with reduced opioid-related mortality, improved treatment retention, and decreased illicit opioid use [11,17,18]. However, although MOUD addresses the physiological aspects of dependence, it may not fully address the psychological and social contributors associated with chronic pain and opioid misuse. Therefore, a holistic biopsychosocial and patient-centered approach that combines pharmacological and non-pharmacological interventions may provide more comprehensive long-term management.

### Methods

A narrative literature review was conducted to examine non-pharmacological and monitoring-based interventions aimed at reducing opioid misuse among adults with chronic pain prescribed opioid therapy. Literature searches were performed using PubMed/MEDLINE, Scopus, and Google Scholar. Search terms included combinations of “chronic pain”, “opioid misuse”, “opioid use disorder”, “cognitive behavioural therapy”, “patient education”, “prescription drug monitoring programmes”, “digital health”, “telehealth”, and “non-pharmacological interventions”.

Studies were included if they focused on adults with chronic non-cancer pain who were prescribed opioids or considered at risk of opioid misuse, and evaluated interventions aimed at reducing opioid misuse, unsafe opioid use, dependence, or opioid-related harm. Studies focusing exclusively on acute pain, pediatric populations, or non-English language publications were excluded. Relevant systematic reviews, observational studies, randomized controlled trials, and policy-based studies were considered.

Evidence was synthesized narratively to identify common intervention strategies, regional approaches, clinical implications, and limitations within the current literature.

## 2. Non-Pharmaceutical Options

### 2.1. Cognitive Behavioral Therapy (CBT)

Cognitive Behavioral Therapy (CBT) is a structured, goal-oriented psychological intervention that aims to identify and modify maladaptive thoughts, emotions, and behaviors that contribute to pain perception, psychological distress, and substance misuse [16,19]. In patients with chronic pain, CBT commonly incorporates components such as cognitive restructuring, coping skills training, relaxation techniques, behavioral activation, stress management, and sleep hygiene education [16,20]. CBT is typically delivered through individual or group sessions and focuses on improving pain coping mechanisms, reducing pain catastrophizing, enhancing self-efficacy, and addressing comorbid anxiety or depression [13,14,20]. In the context of opioid use, CBT may help reduce opioid misuse risk by improving emotional regulation, decreasing reliance on opioids for psychological distress, and promoting healthier long-term pain management behaviors [19,20,21,22,23].

Moore’s secondary analysis of Fiellin’s 24-week randomized control study (2016) compared the efficacy of buprenorphine/naloxone versus buprenorphine/naloxone plus Cognitive Behavioral Therapy (CBT) in the treatment of opioid use disorder. The study found that the experimental group (buprenorphine/naloxone plus CBT) had a better abstinence rate than the control group (buprenorphine/naloxone alone), with a small to medium effect size (Cohen’s d = 0.35) [21,22]. The groups were matched for sex, race, and education, and had similar participant numbers and dropout rates (23 vs. 26 participants, with 48% vs. 54% completion rates) [22]. However, the study’s small sample size may limit the statistical power and generalizability of the findings, as the sample consisted mostly of white participants, with only a small number of Hispanic patients, which may not be fully representative of the broader U.S. population.

Similarly, a 2020 study in Iran compared the effects of brief CBT (four sessions) on mental health outcomes, particularly anxiety and insomnia, in individuals with substance-related disorders. The study found a statistically significant decline in anxiety and insomnia in the intervention group (CBT and medical management), with a 2.85 point drop on the General Health Questionnaire 28, compared to a nonsignificant 0.9 point increase in the control group. These findings further support the role of CBT as an effective non-pharmacological intervention, particularly given the established bidirectional relationship between anxiety, chronic pain, and opioid abuse [23]. However, the study’s small sample size (40 participants) is a limitation, as it may affect the robustness of the findings and their applicability to larger populations.

In a systematic review and meta-analysis conducted in 2010 by Hides et al., it was found that there is limited evidence for the efficacy of CBT or CBT with antidepressants for the population cohort of those who have co-occurring substance abuse disorders and depression [19]. Further, another systematic review from 2018 described that this is a small but statistically significant patient group benefiting from pain reduction for the cohort suffering from chronic pain [20]. These reviews signify that despite all the evidence pointing towards the efficacy of CBT combined treatment, it is important to consider the degree of benefit this approach will provide for patients, especially when considering another issue with CBT, patient compliance, which is seen by the dropout rates, as Fernandez et al. found there was a dropout rate of 15.9% at pretreatment, and 26.2% during treatment [24].

### 2.2. Patient Education

Patient education has proven to be a major factor in combating the opioid crisis and preventing opioid-related hospitalizations, deaths, and misuse. A study conducted in 2016 evaluated the effects of outpatient cancer patients receiving opioid medications for chronic pain, and their caregivers receiving educational programs from trained clinic staff on safe opioid use, storage, and disposal. The study reported that patients who received the educational program before their opioid prescription, compared to patients who did not, were 48% more aware of proper opioid disposal methods, 5% less likely to share their opioids with someone else, 7% less likely to have unused medication at home, and 4% more likely to keep their medicines in a safe place. Patients who received the educational program overall reported less unsafe use of opioids, with a significant 7.6% decrease in unsafe behavior [25]. However, the study had a relatively small sample size of 300, and an unequal number of participants between the control and experimental groups (68 and 232, respectively). Additionally, the study was not fully representative of the broader population of patients receiving opioid prescriptions for chronic pain, as it focused specifically on cancer patients. While the sample is limited, the study clearly shows that patient education—being a low-risk, high-benefit intervention—can contribute vastly to combating the opioid crisis. It fosters a culture of informed and responsible opioid utilization and disposal, thus reducing the likelihood of misuse or abuse [25].

Another study conducted in 2022 examined the impact of patient education on opioid use, storage, and disposal patterns among palliative and chronic non-malignant pain patients. The study found that patients who received educational interventions were 29% more likely never to share their prescription opioids, 69% more aware that one dose could be harmful to someone else, and 86% more likely to dispose of opioids properly. These results reinforce the findings of the previous study and provide additional evidence that patient education improves knowledge and behavior related to opioid handling, even across different patient populations (cancer and non-cancer). This further supports the idea that patient education can play a critical role in reducing opioid misuse and its consequences [26].

A Danish cohort study focusing on chronic opioid use before and after patient education and exercise therapy in knee or hip osteoarthritis patients showed a 10% decrease in opioid use from before to after supervised exercise therapy. However, this decrease was deemed insignificant when regulatory changes in opioid prescribing were considered. This suggests that, while patient education can influence opioid handling habits, the impact on opioid use itself may be limited, especially when other factors like prescribing regulations come into play. Therefore, further research is necessary, as the current literature provides limited insight into how education alone affects opioid use in the context of other regulatory factors [27].

Patient education should be a routine part of chronic pain and opioid management. Education can be delivered through face-to-face counseling, written information leaflets, group sessions, telehealth appointments, video consultations, mobile health applications, SMS reminders, and online CBT-based educational platforms. Key topics should include safe opioid use, dosing, side effects, signs of dependence or overdose, safe storage and disposal, non-pharmacological pain management strategies, and opioid tapering where appropriate [25,26]. Digital and telehealth-based interventions may also improve accessibility, patient engagement, and continuity of care, particularly for patients with limited access to in-person services. Overall, accessible and repeated patient education may help reduce unsafe opioid-related behaviors and support safer long-term pain management [25,26,27].

### 2.3. Continuous Drug Monitoring

The increase in opioid prescriptions for pain has been largely attributed to insurance reimbursement systems that incentivize opioids over alternative pain management options. In decentralized healthcare systems, such as in the United States, physicians have greater autonomy and face fewer regulations regarding opioid prescriptions, compared to nationalized systems like those in Europe. This difference increases the likelihood of misuse and potential deception in the prescription process [26].

An important factor in addressing the opioid crisis is the role of the physician’s prescription. A study conducted by Wen et al. analyzed Medicaid prescription data from 2011 to 2016 and found that, with the use of a Prescription Drug Monitoring Program (PDMP), there were 14.4 opioid prescriptions per quarter per 1000 enrolled patients. In contrast, states without such programs had higher prescription rates. Statistical analysis revealed a significant 8.91% reduction in opioid prescriptions where PDMPs were implemented. Additionally, there was a reduction of 4.16 and 13.24 per quarter per 1000 enrollees in opioid-related inpatient stays and opioid-related emergency department visits, respectively [27]. This study underscores the positive correlation between reduced physician opioid prescriptions and decreased opioid-related harm, highlighting the critical role of PDMPs and physician awareness in combating the opioid crisis.

Prescription Drug Monitoring Programs (PDMPs) are state- or nationally operated electronic databases designed to monitor the prescribing and dispensing of controlled substances, particularly opioids. PDMPs allow healthcare professionals and pharmacists to review a patient’s prescription history, identify high-risk prescribing patterns such as early refill requests, multiple prescribers, or frequent emergency department attendances across different locations, and support safer prescribing practices [28,29]. This may help reduce over-prescribing, identify patients at risk of opioid misuse or dependence, and facilitate earlier clinical intervention. Overall, PDMPs aim to reduce opioid misuse, improve clinical decision-making, and decrease opioid-related morbidity and mortality through ongoing prescription monitoring and early identification of potentially unsafe opioid use [28,29,30].

While Wen’s study offers valuable insights, it has several limitations. The data was not randomized, and variations in state-specific prescription laws complicate the ability to establish a clear causal relationship. Additionally, the study was conducted in the U.S., and without details on patient demographics, the findings may not be generalizable to other countries with different healthcare systems and population profiles.

Further evidence supporting the importance of drug monitoring comes from a 2016 study that analyzed data from The National Ambulatory Medical Care Survey (2001–2010) to compare physician prescription practices before and after the implementation of PDMPs. This study found a statistically significant decrease in the prescription of Schedule II opioids, with a sustained reduction in the first six months following the introduction of these programs [31]. However, since the data is now over a decade old, more recent studies may yield different results, as PDMPs and prescription guidelines have evolved over time.

A 2019 study used fixed effects models to investigate the relationship between PDMP regulatory strength and opioid- and heroin-related deaths from 1999 to 2016. The study found a significant positive correlation between the reduction in opioid prescriptions and a decrease in heroin-related deaths [28]. This suggests that while PDMPs may reduce prescription opioid use, some patients may turn to illicit drugs like heroin as a substitute, introducing new challenges. Additionally, Judd’s literature review found that the effectiveness of PDMPs is inconclusive in rural areas, highlighting that these programs are not universally effective and may have limited impact in certain populations [29].

Despite the conflicting evidence surrounding the effectiveness of PDMPs, it remains a critical non-pharmaceutical approach to combating the opioid crisis. However, its implementation must be handled with caution, especially in regions where its impact may be less significant.

## 3. Gaining Valuable Insights from Regional Approaches to the Opioid Crisis

Although this review focuses on non-pharmacological interventions, it is also important to consider how different regions have approached the opioid crisis using combinations of pharmacological, educational, monitoring, and harm reduction strategies. As the opioid crisis is complex and influenced by differences in healthcare systems, culture, and population needs, no single approach is likely to work for all patients. Examining regional approaches may therefore help identify effective strategies that can be combined into a more holistic, multimodal, and patient-centered approach to chronic pain and opioid misuse management.

### 3.1. North America

The approach to the opioid crisis in North America varies, but a significant focus has been placed on medication-assisted treatment (MAT). In Mexico, MAT is the central policy for addressing opioid use disorder, while Canada has focused on treating marginalized populations, and the United States has worked to increase access to MAT. Despite the emphasis on medical solutions, there are known challenges such as physician over-prescribing, physicians’ low confidence in managing addiction, and inadequate infrastructure for delivering MAT [17]. North America also monitors high-risk behaviors, such as obtaining prescriptions from multiple prescribers, through Prescription Drug Monitoring Programs (PDMPs).

When compared to other detoxification strategies, MAT has shown greater effectiveness in reducing the risk of mortality and morbidity, improving treatment retention, and promoting sustained opioid abstinence. MAT has also been strongly associated with a reduction in the transmission of blood-borne diseases, with a greater than 60% decrease in HCV rates among 552 young adult intravenous drug users (IVDUs) in San Francisco. However, the effectiveness of MAT depends heavily on patient compliance, which remains a significant barrier in addiction treatment. Additionally, MAT is considered a secondary approach rather than a primary one, as it does not directly address the root causes of opioid use disorder [18].

PDMP implementation has been correlated with a decrease in opioid-related deaths and improved physician monitoring. Data show a 22% decrease in opioid prescriptions from 2013 to 2017, with a 9% decrease from 2016 to 2017 alone. Despite reductions in opioid prescriptions and dispensing, opioid-related deaths continue to rise, highlighting that over-prescription is not the sole cause of the opioid crisis [18].

### 3.2. South America

The opioid crisis in South America presents unique challenges compared to other regions. Up until the 1970s, opioid misuse was relatively rare, with notable exceptions in Puerto Rico and Panama. However, opioid dependency rates began to rise in the early 2000s, although not at the same alarming levels seen in North America. Despite South America producing 3% of the world’s morphine and heroin, the region’s opioid consumption remains disproportionately low, raising concerns about potential underdiagnosis of opioid dependency. This discrepancy suggests that many individuals in South America may not be receiving timely diagnoses or adequate treatment. For instance, a study conducted at University Hospital in Medellín, Colombia, found that opioid dependency was often diagnosed too late in a group of 60 patients [32]. Delayed diagnosis and limited access to addiction services may be key contributors to the gap between opioid production and consumption. While this study offers valuable insights into the situation in Colombia, its small sample size and regional focus limit its generalizability. To address the opioid crisis in South America, it will be crucial to improve early diagnosis, expand treatment access, and enhance prevention efforts to prevent further escalation.

In response to growing opioid dependency, Latin America has implemented harm reduction programs. Mexico, in particular, has established clear programs for alternative opioid drug treatments [33]. Other countries like Argentina, Brazil, Paraguay, and Uruguay have created programs to manage synthetic psychoactive drug use, although these programs primarily focus on drugs other than opioids [33]. While South America does not face the same scale of opioid addiction as North America, the existing programs focus on continuous drug prescription monitoring and some alternative therapies. As the opioid crisis continues to evolve in the region, expanding these harm reduction programs and increasing access to opioid-specific treatments will be essential in combating the issue.

### 3.3. Europe

Europe differs in its opioid crisis as it is not as severe as the United States, and it focused on stricter regulation of opioids by having regulatory frameworks of PDMPs and more focus on psychological pain management. The main issue of the opioid crisis in Europe is that the majority of those who abuse opioids remain outside the treatment programs, which is why they focus on efforts to improve primary care risk assessment and interventions to reduce opioid demands [34,35].

Like in North America, PDMPs are used in Europe, yielding similar results in terms of reduced opioid dispensing but not fatalities.

As Europe focuses on prevention, the presence of supervised drug-consumption facilities has demonstrated a decrease in overdoses, promotion of safer injection conditions, enhanced access to primary healthcare, and a 30% increase in the uptake of detoxification treatment. Another advantage of these facilities is the decreased HIV transmission rates due to accessible clean needles [34].

Risk assessment includes the screening and treatment of comorbid psychiatric conditions, as there is an increased risk of developing OUD. Thus, Europe focuses on reviewing past and current psychiatric history before commencing treatment to ensure that comorbid conditions receive appropriate diagnosis, treatment and support, as well to reduce the risk of OUD [34].

### 3.4. Middle East

The opioid crisis in the Middle East is significantly better due to the limited availability of opioids for pain management [36]. Likewise, there is also limited access to medication for opioid use disorders due to financial and geographic barriers [37]. Therefore, in the Middle East, opioid misuse is not as prevalent as the West, and it varies depending on the country, which is why there is no clear treatment approach.

### 3.5. Asia

Most people with opioid dependence in Asia, specifically South Asia, use heroin and opium, mainly through the injection route. Due to the large burden of injection use and the increase in HIV rates, countries in South Asia have well-established needle and syringe campaigns, yet they lack opioid substitution therapy [38]. Anti-addiction programs in Asia vary from region to region, private or public, coercive or voluntary. In Asia, specifically Hong Kong, rehabilitation facilities work with medical assistance, self-help programs and methadone maintenance therapy [39]. Overall, the opioid crisis and prevention in the region are under-researched and underdeveloped.

### 3.6. West Africa

There has been a surge in tramadol abuse in Ghana due to increased consumption for either physical pain or pain due to anxiety. Initially, the approach adopted to manage this crisis was focused on policy and law, but in 2020 it shifted to a harm reduction approach as they signed a bill that views drug misuse as a public health concern rather than a criminal justice problem [40]. This shift will make it easier and more accessible for those who suffer from opioid use disorder to receive treatment.

### 3.7. Australia

As found in a longitudinal study done by Crispin et al., Australia has recently seen a dramatic increase in the use of prescribed opioids for psychological distress and social stressors [41]. As a result of this rise, in 2018 Australia up-scheduled codeine-containing products from over-the-counter access to requiring prescription [42]. Since this change in 2018, Australia has seen a significant reduction in codeine supply, codeine poisoning calls, overdoses, and unnatural deaths [43]. In addition, take-home naloxone programs have successfully been implemented into the healthcare system [44]. To conclude, Australia’s prevention program focuses on up-scheduling codeine-containing drugs.

## 4. Discussion: Our Proposed Approach to the Crisis

By reviewing numerous reports, each looking at the opioid crisis from a different perspective, it becomes evident that it is a complex problem that has multiple causes and secondary issues that arise because of it. Different countries have tried multiple approaches that include some non-pharmaceutical solutions, and upon reviewing the long-term effects and effectiveness of these solutions, it becomes evident that there are repercussions. For example, there was an increase in heroin-related morbidity and mortality due to regulatory laws reducing opioid prescriptions. This shows the importance of viewing the crisis holistically with a long-term outlook.

Non-pharmaceutical solutions have a significant effect in helping reduce the morbidity and mortality of the opioid crisis, but this approach on its own cannot achieve a large reduction in opioid misuse due to many external factors like patient and doctor compliance to programs like CBT, patient education and continuous drug monitoring. Despite this, these non-pharmacological solutions pose no clear risk to patient health, meaning that their potential benefits outweigh any risks and should be incorporated in conjunction with medication therapy.

One question that arises from the use of CBT is its generalizability worldwide due to different cultural sensitivities. However, a systems review done in 2023 by Huey et al. found that CBT is generally effective in ethnic minorities and trends suggesting CBT is weaker for some ethnic groups were found to be insignificant [45]. In addition, there are culturally modified CBT approaches that can be used in different regions that are both effective and culturally acceptable [46]. Overall, CBT is a powerful tool when combating the opioid crisis and should be implemented either in its traditional form or culturally adapted form in all regions, regardless of their cultural sensitivities.

In addition, the focus population in this mini-review are the older age group, who suffer from chronic pain in addition to potential opioid addiction issues. To achieve better outcomes in the opioid crisis, these two conditions must be looked at separately and treated as two different entities with either medication, non-pharmaceutical management or a combination of both.

Furthermore, upon reviewing each region’s successes and failures, we consider that the optimal approach for the opioid crisis should be patient-centered. Each patient should be reviewed for risk factors (as is the case in Europe) and have a management plan catering to their needs. Different people have different personalities and needs, and as previously established, the crisis is multimodal and complex, like patients. Some patients may believe in more natural treatment plans, meaning they will have better compliance with non-pharmaceutical approaches, while others may believe in medical management needing an approach similar to North America’s that heavily focuses on PDMP.

Patient-tailored care that involves all choosing from the previously discussed solutions to the opioid crisis should be the future of the crisis, but there are limited studies on this topic and it should be a new field of focus in opioid research.

## 5. Conclusions

The opioid crisis remains a complex public health issue that requires a multifactorial and patient-centered approach. This review highlights the potential role of non-pharmacological interventions, including cognitive behavioral therapy, patient education, and prescription drug monitoring programs, in reducing opioid misuse risk among adults with chronic pain. While these interventions should not replace pharmacological management or Medication for Opioid Use Disorder (MOUD), their integration into routine care may improve long-term outcomes and reduce opioid-related harm. Where appropriate and acceptable to the patient, non-pharmacological strategies should be considered alongside pharmacological management, with clinicians educating patients on the risks of long-term opioid use and the importance of developing sustainable long-term pain management strategies. Management should remain individualized and tailored to each patient’s age, clinical condition, functional status, and life expectancy.

Different regional approaches demonstrate that no single intervention is universally effective, and management strategies should therefore be adapted to individual patient needs, healthcare systems, and cultural contexts. Clinically, greater emphasis should be placed on holistic pain management, risk assessment, patient education, psychological support, and safe opioid prescribing practices. Further large-scale and longitudinal studies are needed to evaluate the long-term effectiveness, accessibility, and implementation of combined multimodal interventions in reducing opioid-related harm, including the long-term impact of COVID-19-related healthcare changes such as increased tele-prescribing, reduced face-to-face care, and greater reliance on remote interventions.

## Data Availability

No new data were created or analyzed in this study.

## Abbreviations

CBT	Cognitive Behavioral Therapy
HCV	Hepatitis C Virus
HIV	Human Immunodeficiency Virus
IVDU	Intravenous Drug Users
MAT	Medication-Assisted Treatment
MOUD	Medication for Opioid Use Disorder
PDMP	Prescription Drug Monitoring Program
OUD	Opioid Use Disorder
